# Saline Subcutaneous Injections

**Published:** 1890-10-11

**Authors:** 


### SALINE SUBCUTANEOUS INJECTIONS.

We recently reported a case of poisoning by antifebrine, in which the slow subcutaneous injection of half-a-pint of a solution of common salt acted as a powerful stimulant to the heart when injections of ether, alcohol given by the mouth, and other means had given by no means encouraging results. This procedure has rarely been adopted in this country except in the treatment of cholera by Dr. Parkes in India in 1843, by Dr. Stevens in 1854, and by Mr. P. B. Mason and us in 1866, when we used a solution of carbonate of soda and common salt, such as we found patients convalescing from the disease to drink with intense relish, since it restored the salts that had been withdrawn from the blood during the diarrhœa of the algide stage. Dr. Sahli, of Berne, has, however, employed subcutaneous injections of what he calls the "physiological solution," *i.e.*, 0·73 per cent. of sodium chloride, in a number of conditions mostly toxæmic, but also as a substitute for drinking in cases of perforation of, or operations on, the stomach and intestines. He, like other continental physicians, has discarded mechanical injection in favour of simple gravitation from an Erlenmeyer's flask, using a hollow needle about the size of a knitting-pin. With this he can inject as much as a litre in from five to fifteen minutes. The solution should be previously sterilised, and maintained during injection at the normal temperature of the body. The most convenient site for the puncture is the anterior wall of the abdomen, the skin having first been washed with soap and with a sublimate solution. Anæsthesia he rarely finds necessary, and with these precautions, together with the subsequent sealing of the wounds with sterilised cotton-wool and collodion, no signs of local irritation whatever are observed, even if the operation be repeated, as it safely may be, four or five times a-day.

The effects are, he believes, (1) the restoration of normal arterial tension after profuse hæmorrhage, diarrhœa, &c.; (2) the supply of water to tissues in which it has been reduced by these conditions; (3) the dilution of poisons circulating in the blood and lymphatic systems; and (4) their rapid elimination, together with the waste products of metabolism, in consequence of the profuse diuresis and greatly increased excretion of urea, &c., which follows in all cases when the arterial and vascular tension has been restored or in which it has not been previously subnormal.

Thus the injection of a litre twice daily in uræmia complicating acute or chronic nephritis, is speedily followed by an abatement of all the symptoms. In these cases the result is aided and accelerated by the internal administration of digitalis, which, by strengthening the heart's action and increasing arterial tension, gives a stimulus to the elimination of water, urea, &c., and averts any tendency to œdema.

In the "typhoid state" of many diseases injections are followed by cessation of delirium, the pulse becoming stronger and fuller, and the tongue moist.

In cholera, and diarrhœas attended with a great drain of the fluids of the body and collapse, and in cases of acute anæmia from hæmorrhage, the water refills the vessels, restoring their tension, and enabling the blood to circulate in the extremities and on the surface, while the salt acts as a stimulant to the depressed cardiac centres.

In virtue of the effects (3) and (4) it is useful in poisoning, especially with drugs that tend to be eliminated by the kidneys, and it is indicated in perforation of, or operations on the stomach or bowels, in peritonitis, ileus, &c., in which physiological rest of the gastro-intestinal tract is imperative.

The injections in cholera to which we have alluded were intravenous, not subcutaneous, and open to the objections that the vascular system might thus be distended so rapidly as to cause embarrassment of the heart's action, and that the number of great veins available for the purpose was limited, while each venesection was necessarily followed by more or less complete obliteration of the vessel.

In all cases of intravenous injections in cholera egg albumen has been added with the intention of producing a fluid resembling serum in its density and composition. But experiments on dogs, &c., have shown that egg albumen is converted into serum albumen only when slowly introduced into the portal vein so as to be transformed during its passage through the liver. If injected into the systemic vessels it is eliminated unchanged by the kidneys, producing albuminuria; but in cholera, while the excretory functions are in abeyance, the result of such injections can only be the loading of the already concentrated blood with a foreign and worse than useless material. The albumen ought in any future experiments to be omitted unless by the substitution of defibrinated blood or blood serum for the saline solution.

Dr. Dickenson's treatment of cases of nephritis by enormous draughts of water alone, the simple flushing out of the kidneys, as he called it, and without the aid of any drugs until he had satisfied himself as to the correctness of his principle, the results of which gave the death-blow to the old and unscientific dosing with hydragogue cathartics, was in fact an anticipation of the treatment now recommended by Professor Sahli, though the means employed to attain the same end were different and simpler.

